# Identification and verification of the temozolomide resistance feature gene DACH1 in gliomas

**DOI:** 10.3389/fonc.2023.1120103

**Published:** 2023-03-07

**Authors:** Qiang Gu, Lang Li, Jiahao Yao, Fa-yan Dong, Yifan Gan, Shuhuai Zhou, Xinyu Wang, Xue-feng Wang

**Affiliations:** Harbin Medical University, Harbin, China

**Keywords:** glioma, temozolomide, drug-resistance, risk model, machine learning

## Abstract

**Introduction:**

The most important chemotherapy treatment for glioma patients is temozolomide. However, the development of drug resistance severely restricts the use of temozolomide. Therefore, elucidating the mechanism of temozolomide resistance, enhancing temozolomide sensitivity, and extending patient survival are urgent tasks for researchers.

**Methods:**

Temozolomide resistance hub differential genes were identified using differential analysis and protein interaction analysis from the GEO datasets (GSE100736 and GSE113510). These genes were further studied in glioma patients treated with temozolomide in the TCGA and CGGA databases. Patients from the mRNAseq_325 dataset (CGGA) were considered as the training set to construct a risk model for predicting glioma sensitivity to temozolomide, while patients from the mRNAseq_693 dataset (CGGA) and TCGA-GBM dataset were considered as the validation set to evaluate the performance of models. PCR and western blot were performed to determine the difference in expression of the feature gene DACH1 between glioma cells and temozolomide-resistant glioma cells. The alterations in the sensitivity of tumor cells to temozolomide were also observed after DACH1 was silenced. The patients were then divided into two groups based on the expression of DACH1, and the differences in patient survival rates, molecular pathway activation, and level of immune infiltration were compared.

**Results:**

Based on four signature genes (AHR, DACH1, MGMT, and YAP1), a risk model for predicting glioma sensitivity to temozolomide was constructed, and the results of timeROC in both the training and validation sets showed that the model had good predictive performance. The expression of the signature gene DACH1 was significantly downregulated in temozolomide-resistant cells, according to the results of the PCR and western blot experiments. The sensitivity of tumor cells to temozolomide was significantly reduced after DACH1 was silenced. DACH1 probably regulates temozolomide resistance in glioblastoma through the transcriptional dysregulation in cancer and ECM.

**Discussion:**

This study constructs a risk model that can predict glioma susceptibility to temozolomide and validates the function of the feature gene DACH1, which provides a promising target for the research of temozolomide resistance.

## Introduction

Gliomas are the most common primary intracranial tumors, which mostly affect the frontal and temporal lobes of the cerebral hemispheres (1). Although this tumor shares many characteristics with typical glial cells, its precise origin is yet unknown (2). The current standard of care is surgical resection combined with postoperative radiation and temozolomide chemotherapy, followed by continued adjuvant chemotherapy with temozolomide (3). Despite the benefit of standard treatment, the prognosis for patients is still poor, with a median survival rate of less than 15 months and a five-year survival rate of only 6.9% (4). This is closely related to a number of factors, such as the tumor’s infiltrative growth, the inadequate surgical resection, and the development of chemo-radiotherapy resistance (5). Temozolomide is a novel, 2nd-generation oral alkylating agent that has been approved by the FDA (Food and Drug Administration) for the treatment of brain tumors since 2005 (6). Temozolomide does not directly inhibit the growth of tumors. It can degrade into MTIC (5-(3-methyl-1-triazeno) imidazole-4-carboxamide), and then degrade into AIC (4-amino-5-imidazole-carboxamide) and methane-diazonium. Methane-diazonium is considered as an active alkylating substance that can alkylate the N^7^ and O^6^ sites of guanine and the N^3^ site of adenine, interfering with DNA replication through the MMR (mismatch repair) pathway to exert cytotoxic effects and induce apoptosis in tumor cells (7). Due to its lipophilic nature, temozolomide easily penetrates the BBB (blood-brain barrier) (8). Temozolomide is the recommended chemotherapeutic agent for glioma since it has a good oral absorption ratio and fewer side effects compared to other chemotherapy drugs (such as procarbazine, lomustine, vincristine, etc.) (9). However, as treatment proceeds, tumors gradually develop chemoresistance, which ultimately results in treatment failure. Currently, the theoretical explanations for Temozolomide resistance include DNA repair mechanisms, survival autophagy, glioma stem cells (GSCs), drug efflux transporters, and others (10). However, the precise mechanisms through which drug resistance develops are still unknown.

Machine learning is a multidisciplinary technique that combines statistics and computer science and uses a variety of strategies and algorithms to arrive at the best model (11). Compared with traditional statistical methods that concentrate on the causality of hypothesis testing and the significance of model features, machine learning focuses more on the downscaling of high-dimensional data and the predictive performance and generalization of models (12, 13). As a result, machine learning is better suited for analyzing complex and large quantities of data (e.g., gene expression analysis, image feature extraction, drug sensitivity prediction, etc.).

In conclusion, we developed a risk model to predict the prognosis of patients treated with temozolomide using gene expression profiles and clinical data from the GEO (Gene Expression Omnibus), TCGA (The Cancer Genome Atlas), and CGGA (Chinese Glioma Genome Atlas) databases. One of the model’s signature genes (DACH1) was identified and validated, providing a promising target for temozolomide resistance research.

## Materials and methods

### Patients and datasets

The RNA expression profiles of glioma cells and glioma TR (temozolomide-resistance) cells were obtained from the GEO database. There was a total of two datasets: GSE100736 contained the RNA expression profiles of three U251 and three U251TR, while GSE113510 contained the RNA expression profiles of three LN229 and three LN229TR. The RNA expression profiles and clinical information of glioma patients were obtained from the CGGA and TCGA databases. The training set included 188 patients from the mRNAseq 325 dataset (CGGA), the validation set I included 435 patients from the mRNAseq_693 dataset (CGGA), and the validation set II included 58 patients from the TCGA-GBM. Selection criteria include: 1. receiving standard temozolomide treatment; 2. having a survival time of more than 30 days; 3. having a glioblastoma with a clear pathologic diagnosis. R software (version 4.2.0) was used to standardize, batch-correct, quality-check, and ID-transform the data.

### Differential expression analysis

Differential expression analysis was performed using the “limma” package (version 3.52.1) for microarrays from the GEO database and the “DESeq2” package for RNA-seq from the CGGA and TCGA databases in R software. Volcano plots were used to depict differential expression genes (DEGs).

### Protein-protein interaction

DEGs were imported into the string website (14) (https://cn.string-db.org/) to build PPI networks online. The generated networks were then imported into the Cytoscape software (version 3.9.1) to analyze the hub node of the network using the Centiscape plugin, with the following selected parameters: degree, betweenness, Eigenvector centrality, and bridging centrality. For the genes whose scores were all above 50%, we defined them as hub DEGs.

### Model construction and evaluation

A batch univariate COX regression analysis was conducted to identify genes that were significantly related with patient prognosis (p <0.05). Genes that were risky for prognosis and up-regulated in the DEGs as well as genes that were protective for prognosis and down-regulated in the DEGs were picked out. Then, lasso regression analysis and random forest were used to further screen these genes. The risk model was built using the final feature genes that had been screened, and the patient’s riskScore values were calculated (riskScore = h_0_ (t)*exp(β1x1+β2x2+… + βnxn). The performance of the model was evaluated using the training set and validation set data.

### Drug prediction

Based on the riskScore values calculated by the model, patients were divided into high- and low-risk groups, and differences in temozolomide sensitivity between high- and low-risk groups were predicted by the “oncoPredict” package. Drugs that can reverse feature gene expression were predicted online with the help of the SPIED3 website (http://212.48.67.52/cgi-bin/HGNC-SPIED3.cgi).

### Functional prediction of DACH1

Genes that interact with DACH1 were identified on the GeneMANIA website (http://genemania.org/), and these genes were entered into the Metascape website (https://metascape.org/gp/index.html#/main/step1) for enrichment analysis of GO biological processes and KEGG pathways to predict the function of DACH1.

### Cell culture and inducing temozolomide-resistant clones

U251 human-derived GBM cell lines were obtained from the Chinese Academy of Sciences Cell Bank and cultured using 90% DMEM (Dulbecco’s modified Eagle medium, Gibco) + 10% FBS (fetal bovine serum, Hyclone) at 37° and 5% CO2 environment. After the cell proliferation was stabilized, ten U251 clones were selected for the construction of drug-resistant cell lines by the drug concentration increment method. The temozolomide concentrations were in the following order: 0.125 μg/mL, 0.25 μg/mL, 0.5 μg/mL, 1 μg/mL, 2 μg/mL, 4 μg/mL, 8 μg/mL, and 16 μg/mL. Each concentration was maintained until the cells could keep growing steadily for five to six generations. After more than 6 months of induction, three U251 clones were successfully converted to U251TR, which were employed in subsequent biological experiments.

### Cell transfection

The DACH1 shRNA lentiviral vector was purchased from Shanghai Genechem Co., Ltd. The interference target sequence is 5’-GCACTTGAGTTTGAGACGA-3’ [from the research by Zhao et al. (15)]. The vector component is hU6-MCS-CBh-gcGFP-IRES-puromycin. U251 cells were cultured in 6-well plates at 10,000 cells per well, incubated for 24 h, and then replaced with medium containing virus (MOI = 5) for transfection, which took roughly 8 h. Puromycin (concentration: 2 μg/ml) was then used to further treat U251 cells for 48 hours in an effort to remove any failed transfections.

### Quantitative real-time PCR

RNAiso Plus (Takara, Code No. 9108) and PrimeScript™ RT reagent Kit (Takara, Code No. RR047A) were used to extract and reverse-transcribe total RNA to cDNA. The SYBR Green Premix Ex Taq kit (Takara, Code No. RR820A) was used to perform real-time quantitative fluorescence analysis in the CFX Connect Real-Time System (Bio-Rad, USA). The experimental steps are followed according to the manual. The PCR procedure was 95°C for 30 s, 45 cycles at 95°C for 10 s, and 55°C for 30 s. Relative mRNA expression was calculated through the 2^-ΔΔCT^ method and adjusted to GAPDH. Prism software was used to analyze and display the results. The primer sequences are as follows: GAPDH (forward primer: 5’- AGTAGAGGCAGGGATGATG-3’; reverse primer: 5’- TGGTATCGTGGAAGGACTC-3’); DACH1 (forward primer: 5’- GGAATGGATTGTGGCTGAAC-3’; reverse primer: 5’- GGTATTGGACTGGTACATCAAG-3’).

### Western blot

Cell lysis buffers (Beyotime) were used to lyse the cell. Protein concentrations were determined by the BCA method and finally diluted to 3 μg/μl. Electrophoresis was performed using 12.5% PAGE gel with a loading volume of 10 μl per lane. After electrophoresis, proteins were transferred from gel to PVDF membrane. The PVDF membrane was sealed for 2 h with 5% skim milk, then incubated with primary antibodies at 4°C for 12 h and secondary antibodies at room temperature for 1.5 h. Prior to each step, the membrane was washed three times with TBST for ten minutes. Final imaging by ECL (enhanced chemiluminescence). Both primary and secondary antibodies were purchased from the Proteintech company. Primary antibodies included DACH1 monoclonal antibody (Cat No: 60082-1-Ig, dilution: 1:5000) and GAPDH monoclonal antibody (Cat No: 60004-1-Ig, dilution: 1:50000). The secondary antibody is HRP-conjugated Affinipure Goat Anti-Mouse IgG(H+L) (Cat No: SA00001-1, dilution: 1:5000).

### Temozolomide toxicity experiment

Cells from the experimental and control groups were cultured in 96-well plates with 5000 cells per well for 24 h and then replaced with serum-free medium containing different concentrations of temozolomide (GLPBIO) (0 μM, 400 μM, 1000 μM, 1600 μM, 2200 μM, 2800 μM in order). The CCK8 (GLPBIO) (final concentration of 10%) was administered after 48 h of incubation. The OD value was measured after 1 hour, and the cell survival rate was calculated. Cell survival rate = (experimental group - blank group)/(control group - blank group). The IC50 was extrapolated from a fitted curve made using the cell survival rate and temozolomide concentrations.

### Scratch assay

Cells were cultured in 6-well plates with 30,000 cells per well after marking the 6-well plates’ bottom surface. After 24 h, the 6-well plates were scratched perpendicularly to the marker line and replaced with medium containing temozolomide (concentration: 50 μM). The marker points at 0 and 24 hours, respectively, were captured on camera. The formula for calculating the cell migration rate is (1-24 h scratch area/0 h scratch area) * 100%.

### Survival analysis

Based on the feature genes’ median levels of expression, the samples were divided into two groups. The “survival” package was used to analyze survival differences between the two groups.

### Analysis of immune infiltrates and gene set enrichment analysis

Based on the median value of the feature gene DACH1 expression, patients in the training set were divided into high and low expression groups, and differential expression analysis was run for the two groups. Genes were sorted according to logFC value from largest to smallest, and GESA was performed by the “clusterprofiler” package to find potential molecular pathway associations for the high and low expression groups. Pathways with a P-value < 0.05 were considered statistically significant. The “Estimate” package was applied to examine the immune score and tumor purity of patients. The Cibersortx website (https://cibersortx.stanford.edu/) (16) was visited to examine immune cell infiltration in patients.

### Statistical analysis

The Prism software (version 9.3) and R software (version 4.2) were used for statistical analysis of the experimental results. The t-test was used to compare two groups, and double-factor variance analysis was used to compare several groups. The Kaplan-Meier method was used to compare the survival rates of high-risk and low-risk groups. Risk models were constructed by multivariate Cox regression analysis. In the figure, 'ns' indicates P > 0.05, '*' indicates P ≤ 0.05, '**' indicates P ≤ 0.01, '***' indicates P ≤ 0.001, and '****' indicates P ≤ 0.0001. The prediction accuracy of the risk model was evaluated by TimeROC. There were at least three successful replications in each experiment.

## Result

### Identification of hub DEGs

**Figure 1:**
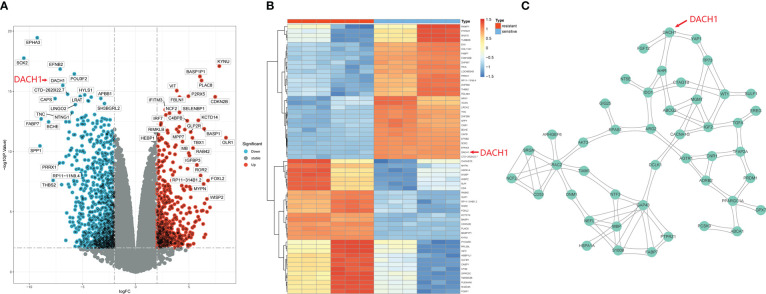
Identification of hub DEGs. **(A)** DEGs are shown by the volcano plot. Red dots represent genes that are upregulated in the resistance group (logFC > 2 and P.value < 0.01), and green dots represent genes that are downregulated (logFC < 2 and P.value < 0.01). **(B)** The heatmap shows the top 30 up-regulated and top 30 down-regulated genes in the drug resistance group. Red means genes up-regulated and blue means genes down-regulated. **(C)** 45 hub DEGs PPI network.

1529 DEGs were yielded by differential analysis from the GSE100736 dataset and the GSE113510 dataset, of which 556 DEGs were up-regulated and 973 DEGs were down-regulated. The results are shown by volcano plots (**Figure 1A**) and heat map (**Figure 1B**). In the volcano plot, the red dots represent genes that are upregulated in the temozolomide resistance group (logFC > 2 and P.value < 0.01) while the green dots represent genes that are downregulated (logFC < 2 and P.value < 0.01) in that group. The heatmap displays the top 30 genes that were up- and down-regulated in the temozolomide resistance group. Blue indicates down-regulated genes, while red indicates up-regulated genes. These DEGs were input into the string website for the PPI network’s construction. 45 hub DEGs network were identified and constructed with the help of the Centiscape plugin of the Cytoscape software (**Figure 1C**).

### Construction and evaluation of a risk model for predicting glioma sensitivity to temozolomide

**Figure 2:**
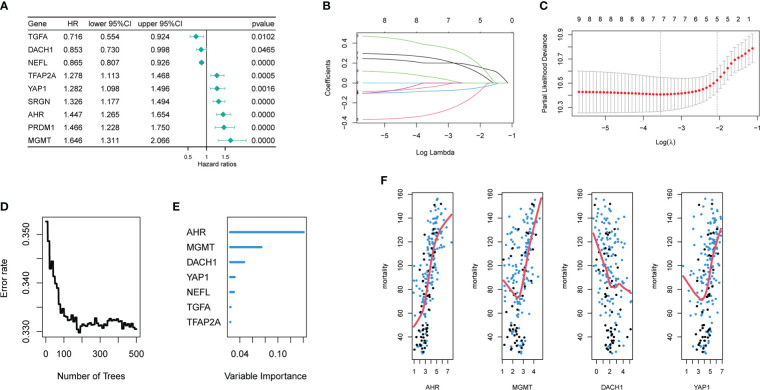
Acquisition of signature genes and construction of risk models. **(A)** Nine genes were significantly associated with the prognosis of temozolomide-treated patient, among which the HR values of TGFA, DACH1, and NEEL were less than 1, and they played a protective role, while the HR values of TFAP2A, YAP1, SRGN, AHR, PRDM1, and MGMT were more than 1, and they played a risky role. **(B)** The nine different colored lines represent nine different variables. As log(λ) increases, the regression coefficients of variables start to converge, eventually reaching 0. **(C)** As the variables gradually converge, the partial likelihood deviance changes in a waveform. When the number of variables is 7, the model performs best. When the number of variables is 5, the model is the simplest. **(D)** As the number of decision trees increases, the error rate gradually decreases. **(E)** Variable importance ranking in random forests **(F)** The top 4 variables of importance in the random forest. (The x-axis represents gene expression level, the y-axis represents mortality, and the red line is the fitted curve.).

**Figure 3:**
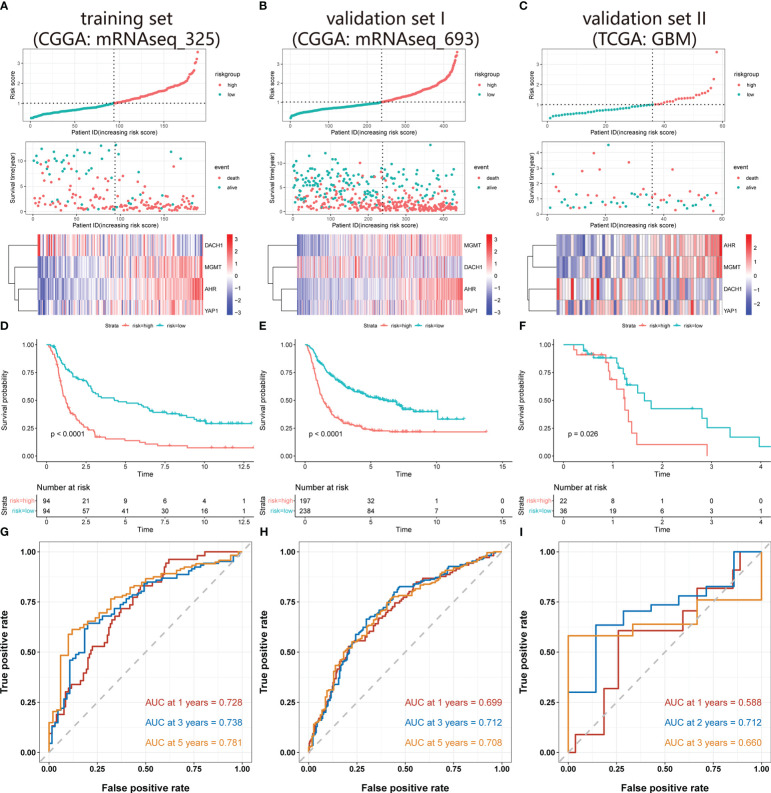
Validation of the risk model. **(A-C)** The top 3 panels show the proportional relationship between the high-risk and low-risk groups. Patients are ranked according to their riskScore from low to high, and the dotted line divides patients into high-risk group (indicated by red dots) and low-risk group (indicated by green dots). The middle 3 panels show the relationship between the riskScore and patient survival time (red dots indicate dead patients, green dots indicate alive patients). As the riskScore increases, the survival time of patients decreases and the number of deaths increases. The bottom 3 panels show the relationship between the riskScore and the expression level of the feature genes. As the riskScore increases, the expression of MGMT, AHR, and YAP1 increases, while the expression of DACH1 decreases. **(D-F)** The red represents the high-risk group, and the green represents the low-risk group. Patients’ survival rates in the high-risk group were significantly lower than in the low-risk group (P<O.O5). **(G–I)** ROC curves and AUC values at different time points. The red, blue, and orange lines in Figures A and B indicate the ROC curves for 1, 3, and 5 years, and in Figure C, the ROC curves for 1, 2, and 3 years. The AUC values are 0.728, 0.738, and 0.781 in the CGGA-mRNAseq_325, 0.699, 0.712, and 0.708 in the CGGA-mRNAseq_693, 0.588, 0.712, and 0.66 in the TCGA-GBM.

Univariate COX regression analysis identified 18 genes from 45 hub DEG that were significantly (P<0.05) related with patient prognosis (**Supplementary material 1**). Finally, nine genes were determined by intersecting genes with HR > 1 and upregulated DEGs as well as genes with HR < 1 and downregulated DEGs (**Figure 2A**). The lasso regression analysis was ran using the “glmnet” package. As log(λ) increases, the regression coefficients of variables start to converge, eventually reaching 0 (**Figure 2B**). As the variables gradually converge, the partial likelihood deviance changes in a waveform. When there are 7 variables, the model performs best (**Figure 2C**). Hence, these 7 genes were chosen for further analysis. The random forest algorithm was executed using the “randomForestSRC” package. As the number of decision trees increased, the model’s error rate gradually reduced (**Figure 2D**). The variables were ordered from high to low importance in the model (**Figure 2E**). The top 4 genes in terms of importance were used as the feature variables (**Figure 2F**), and the coxph function of the “survival” package was used to construct the risk model. In the risk model, riskScore = exp (0.26495 * AHR +0.30219 * MGMT-0.08143 * DACH1+0.07468 * YAP1) *h0(t). We calculated the riskScore of each patient in the training and validation sets based on this formula, then divided the patients into high-risk and low-risk groups based on the median riskScore in the training set. The risk distribution of patients and the expression of feature genes are shown in **Figures 3A-C**. The top 3 panels in **Figures 3A-C** show the proportional relationship between the high-risk and low-risk groups. Patients are ranked from low to high according to their riskScore, and the dotted line divides them into high-risk group (indicated by red dots) and low-risk group (indicated by green dots). The middle 3 panels in **Figures 3A-C** show the relationship between the riskScore and patient survival time (red dots indicate dead patients, green dots indicate alive patients). As the riskScore increases, the survival time of patients decreases and the number of deaths increases. The bottom 3 panels in **Figures 3A-C** show the relationship between the riskScore and the expression of the feature genes. As the riskScore increases, the expression of MGMT, AHR, and YAP1 increases, while the expression of DACH1 decreases. The Kaplan-Meier method was used to determine the difference in survival between the high-risk and low-risk groups. The results show that the survival rates of patients were significantly lower in the high-risk group than those in the low-risk group (P < O.O5) (**Figures 3D–F**). ROC curves were plotted by the “timeROC” package to evaluate the performance of the model. The AUC values for 1, 3, and 5 years were 0.728, 0.738, and 0.781 in CGGA-mRNAseq_325 (**Figure 3G**) and 0.699, 0.712, and 0.708 in CGGA-mRNAseq_693 (**Figure 3H**). Because the patient survival in the TCGA-GBM was all less than 5 years, we calculated AUC values for 1, 2, and 3 years, and they were 0.588, 0.712, and 0.66, respectively (**Figure 3I**). The results showed that the model performed well in terms of prediction.

### Drug prediction

**Figure 4:**
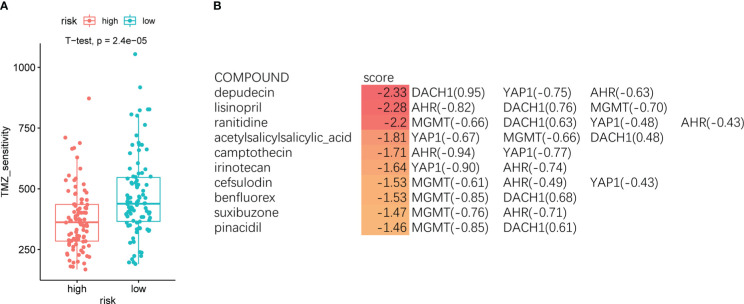
**(A)** The red indicates the high-risk group, and the green indicates the low-risk group. Patients in the high-risk group had significantly lower temozolomide sensitivity than the low-risk group (P < O.O5). **(B)** The top 10 compounds predicted by the SPIED3 website that reversed feature gene expression.

Patients were divided into high- and low-risk groups based on the median riskScore, and the “oncoPredict” package was used to forecast the temozolomide sensitivity. The results showed that patients in the high-risk group had significantly lower temozolomide sensitivity than those in the low-risk group (**Figure 4A**). This result further demonstrated the accuracy of the model. We entered the four feature genes and their logFC into the SPIED3 website to seek some drugs that could reverse the expression of the feature genes. Finally, some compounds were found that were considered to be promising treatments for temozolomide resistance (**Supplementary Material 2**). The top 10 compounds are shown in **Figure 4B**.

### Functional prediction of DACH1

**Figure 5:**
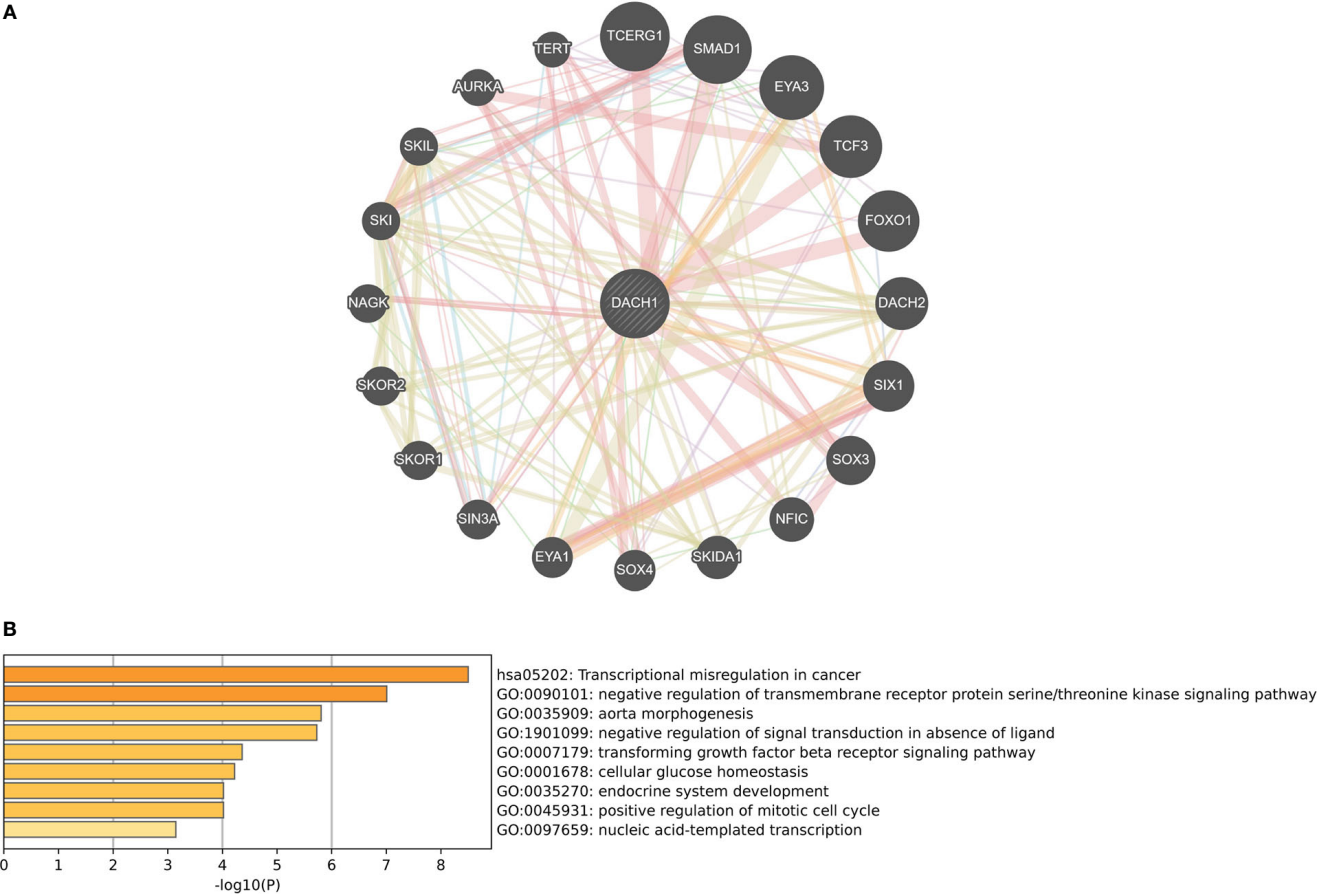
**(A)** Twenty genes that interact with DACH1 were identified on the GeneMANIA website. **(B)** Genes interacting with DACH1 were enriched for functions such as transcriptional misregulation in cancer, TGF-beta receptor signaling pathway, and negative regulation of signal transduction in absence of ligand.

The GeneMANIA website identified 20 genes that interact with DACH1 (**Figure 5A**). Enrichment analysis revealed that genes interacting with DACH1 were enriched for functions such as transcriptional misregulation in cancer, TGF-beta receptor signaling pathway, and negative regulation of signal transduction in absence of ligand (**Figure 5B**).

### Construction and validation of U251TR cells

**Figure 6:**
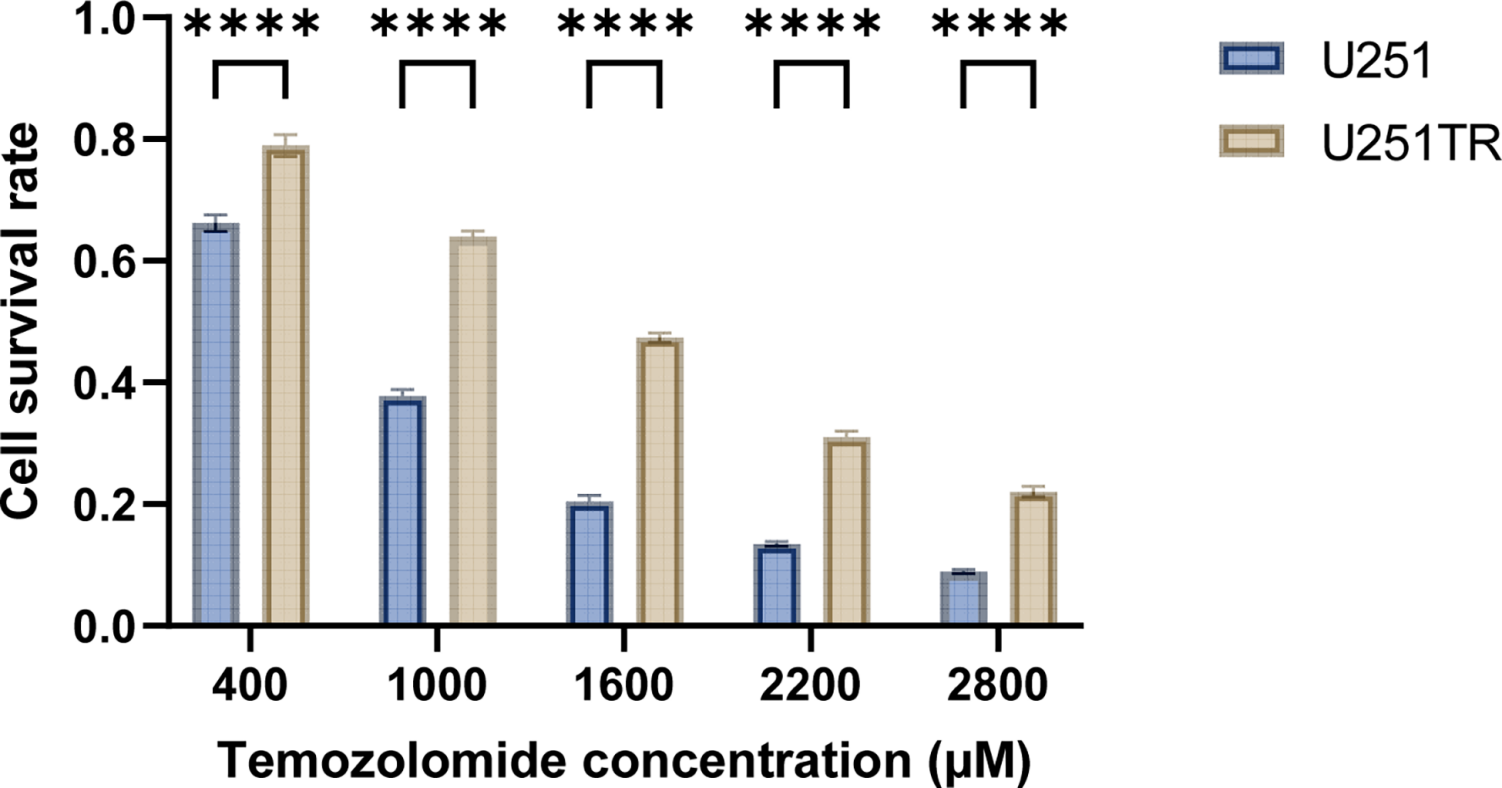
The blue color indicates the U251 group, and the coffee color indicates the U251TR group. The results of temozolomide toxicity experiments revealed that the survival rate of U251 was significantly lower than that of U251TR at various temozolomide concentrations (P<0.05).

After 6 months of continuous induction, 3 out of 10 U251 clones were successfully converted into U251TR. The drug toxicity test indicated that the cell survival rate of U251TR was significantly higher than that of U251 at various Temozolomide concentrations (**Figure 6**). The IC50 was estimated from a fitted curve made using the cell survival rate and temozolomide concentrations. The results suggest that the IC50 of U251TR (1545.45 μM) was about 2.3 times higher than that of U251 (671.8 μM).

### The expression differences of the DACH1 between the U251 and the U251TR

**Figure 7:**
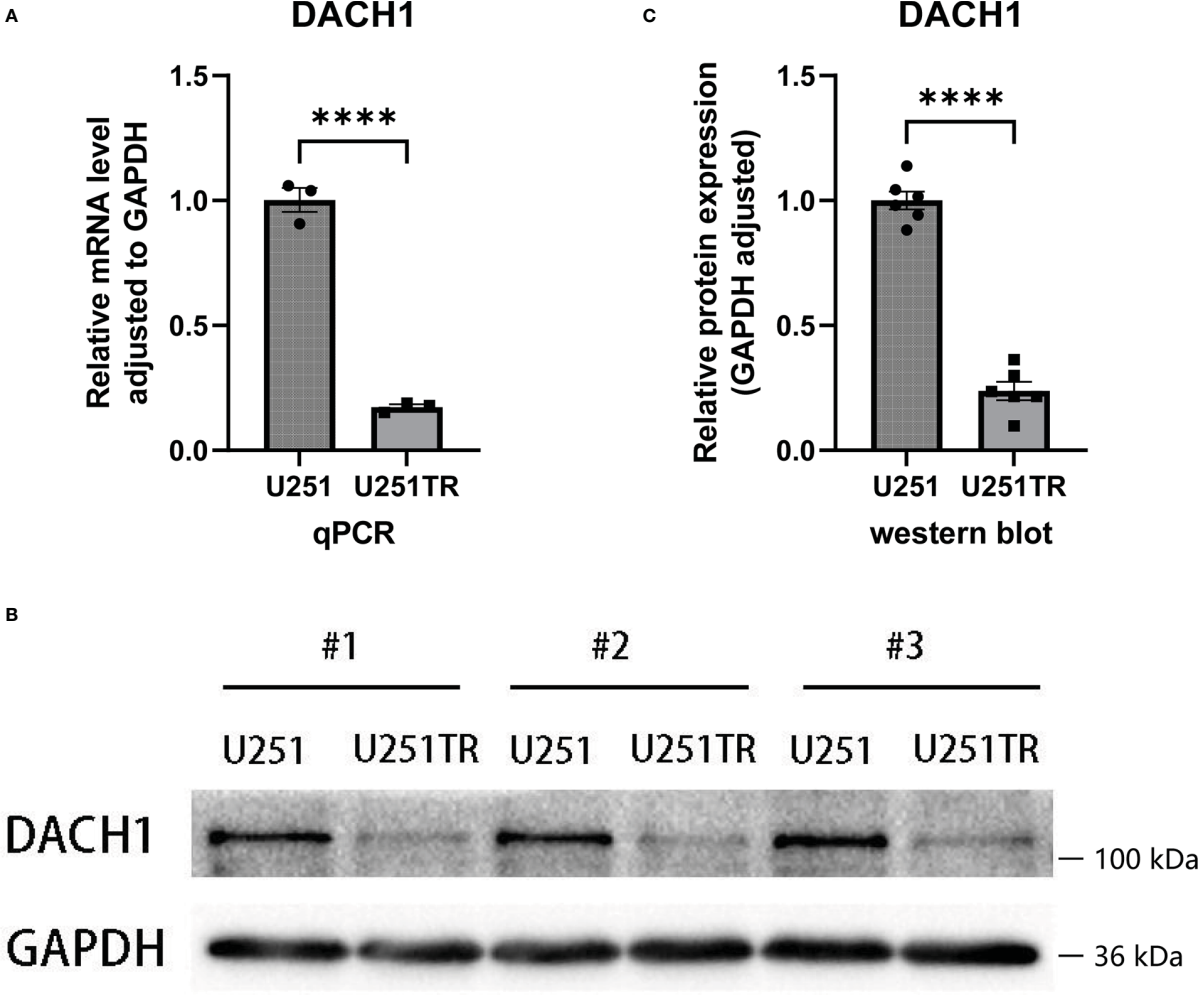
**(A)** The results of PCR experiments indicated that the relative RNA expression of DACH1 (adjusted to GAPDH) was significantly higher in the U251 group than in the U251TR group (P < 0.05). **(B)** The results of western blot experiments indicated that the gray degree of DACH1 was significantly higher in three U251 clones than in three U251TR clones. **(C)** The statistical results of the gray values show that the relative protein expression of DACH1 (adjusted to GAPDH) was significantly higher in the U251 group than the U251TR group.

The qPCR and western blot experiments were performed to determine the difference in DACH1 expression between U251 and U251TR. The results of PCR experiments indicated that the relative RNA expression of DACH1 (adjusted to GAPDH) was significantly higher in the U251 group than that in the U251TR group (P < 0.05) (**Figure 7A**). The results of western blot experiments indicated that the gray degree of DACH1 was significantly higher in three U251 clones than that in three U251TR clones (**Figure 7B**). The statistical results of the gray values show that the relative protein expression of DACH1 (adjusted to GAPDH) was significantly higher in the U251 group than that in the U251TR group (**Figure 7C**).

### The effect of DACH1 on temozolomide sensitivity

**Figure 8:**
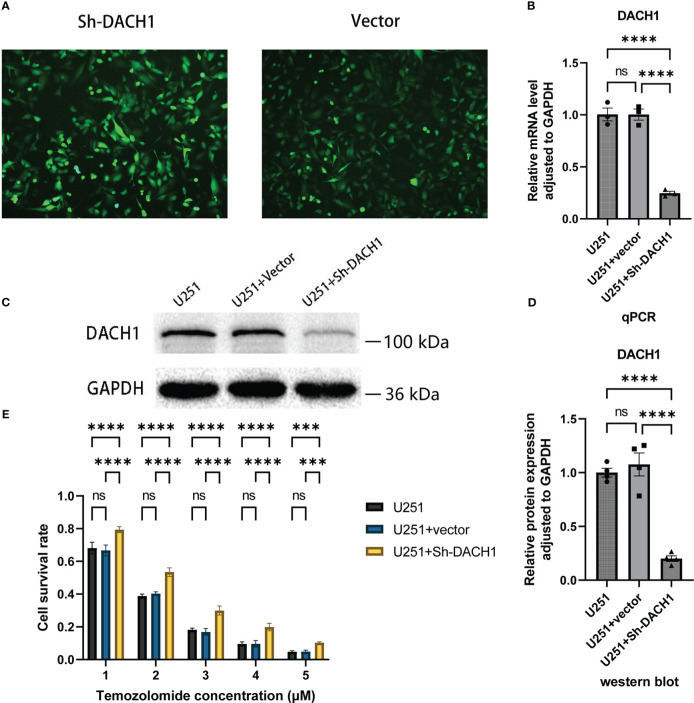
**(A)** The U251 cells in the Sh-DACH1 group and the Vector group display green fluorescence after successful transfection. **(B)** The results of PCR experiments showed that the relative RNA expression of DACH1 (adjusted to GAPDH) was significantly lower in the U251+Sh-DACH1 group than in the U251 group and the U251+Vector group (P < 0.05). **(C)** The results of western blot experiments indicated that the gray degree of DACH1 was significantly lower in the U251+Sh-DACH1 group than in the U251 group and the U251+Vector group. **(D)** The statistical results of the gray values show that the relative protein expression of DACH1 (adjusted to GAPDH) was significantly lower in the U251+Sh-DACH1 group than in the U251 group and the U251+Vector group. **(E)** The result of temozolomide toxicity experiments showed that the survival rate of the Sh-DACH1 group was significantly higher than that of the U251 group and the U251-vector group at different concentrations of temozolomide (P < 0.05).

The expression of DACH1 in U251 was silenced using the lentivirus. Cells display green fluorescence after successful transfection (**Figure 8A**). The effect of silencing was investigated using qPCR and western blot experiments. The results of PCR experiments showed that the relative RNA expression of DACH1 (adjusted to GAPDH) was significantly lower in the U251+Sh-DACH1 group than that in the U251 group and the U251+Vector group (P<0.05) (**Figure 8B**). The results of western blot experiments indicated that the gray degree of DACH1 was significantly lower in the U251+Sh-DACH1 group than that in the U251 group and the U251+Vector group (P<0.05) (**Figure 8C**). The statistical results of the grayscale values show that the relative protein expression of DACH1 (adjusted to GAPDH) was significantly lower in the U251+Sh-DACH1 group than that in the U251 group and the U251+Vector group (P<0.05) (**Figure 8D**). These results suggest that lentiviruses successfully downregulate DACH1 expression. We conducted temozolomide toxicity experiments to discover the effect of DACH1 on temozolomide sensitivity. The results show that the survival rate of the Sh-DACH1 group was significantly higher than that of the U251 group and the U251-vector group at different concentrations of temozolomide (P < 0.05) (**Figure 8E**), indicating that downregulation of DACH1 increases the resistance of U251 to temozolomide.

### Relationship between silencing of DACH1 and cell invasion

**Figure 9:**
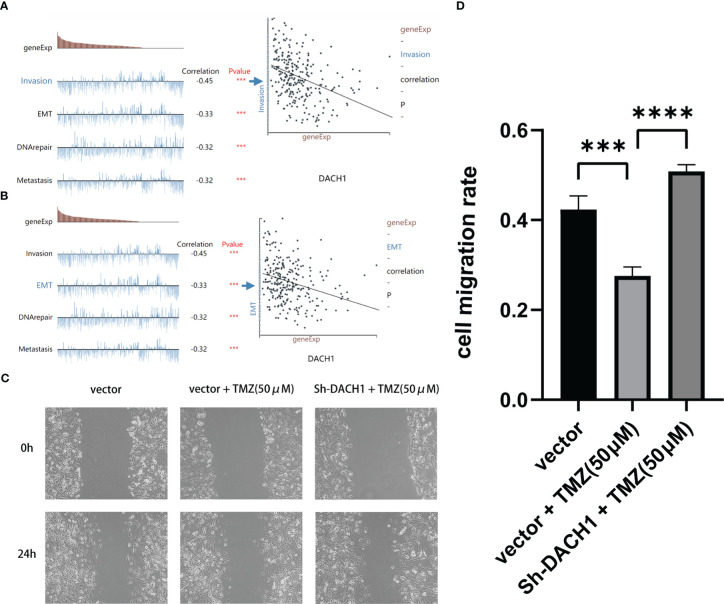
**(A, B)** The cancerSEA website predicts that the lower the expression of DACH1, the stronger the function of invasion and EMT in GBM. **(C, D)** The results of scratch experiments show that the cell migration rate was significantly lower in the vector+TMZ (50 μM) group than in the vector group (P < 0.05) and significantly higher in the Sh-DACH1+TMZ (50 μM) group than in the vector+TMZ (50 μM) group (P < 0.05).

Based on predictions from the cancerSEA website, DACH1 expression was significantly negatively correlated with the function of invasion and EMT in GBM (**Figures 9A, B**). Scratch experiments were performed to observe the effect of DACH1 on the migratory ability of cells. We found that temozolomide at the 50 μM concentration had no effect on U251 proliferation (**Supplementary Material 3**), but significantly inhibited the U251 migration. So, we carried out the scratch assay with temozolomide at a concentration of 50 μM. The results of scratch experiments show that the cell migration rate was significantly lower in the vector+TMZ (50 μM) group than that in the vector group (P < 0.05) and significantly higher in the Sh-DACH1+TMZ (50 μM) group than that in the vector+TMZ (50 μM) group (P < 0.05) (**Figures 9C, D**). According to the results, temozolomide limited U251 migration at the concentrations of 50 μM. However, the inhibitory ability of temozolomide was lost when DACH1 was downregulated.

### The relationship between DACH1 expression and molecular pathways

**Figure 10:**
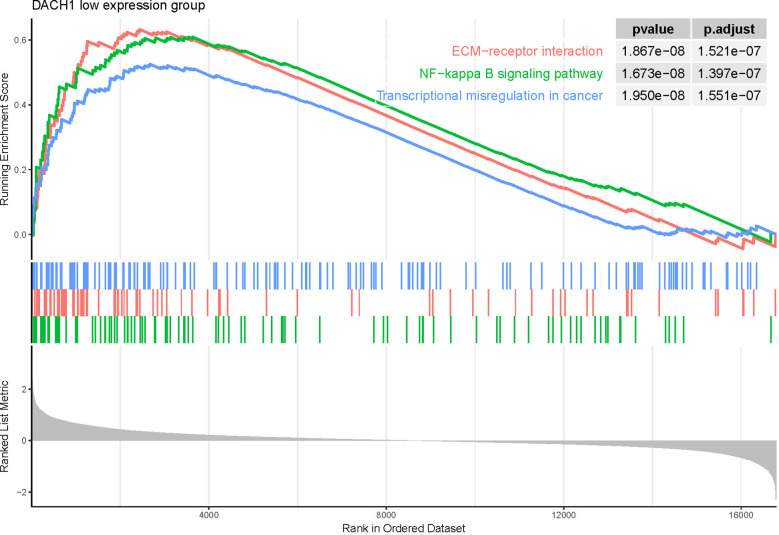
GSEA results showed that the three pathways—transcriptional misregulation in cancer, ECM-receptor interaction, and the NF-kappa B signaling pathway—were activated in the DACH1 low expression group.

Patients treated with temozolomide were divided into high- and low-expression groups based on the median expression of DACH1. Differential expression genes were identified by differential analysis and employed for GSEA. The GSEA results showed that several pathways were activated in the DACH1 low expression group (**Supplementary Material 4**), such as transcriptional misregulation in cancer, ECM-receptor interaction, and the NF-kappa B signaling pathway (**Figure 10**). This result suggests that DACH1 may regulate the sensitivity of gliomas to temozolomide *via* these molecular pathways.

### The relationship between the expression of DACH1 and the immune infiltration status

**Figure 11:**
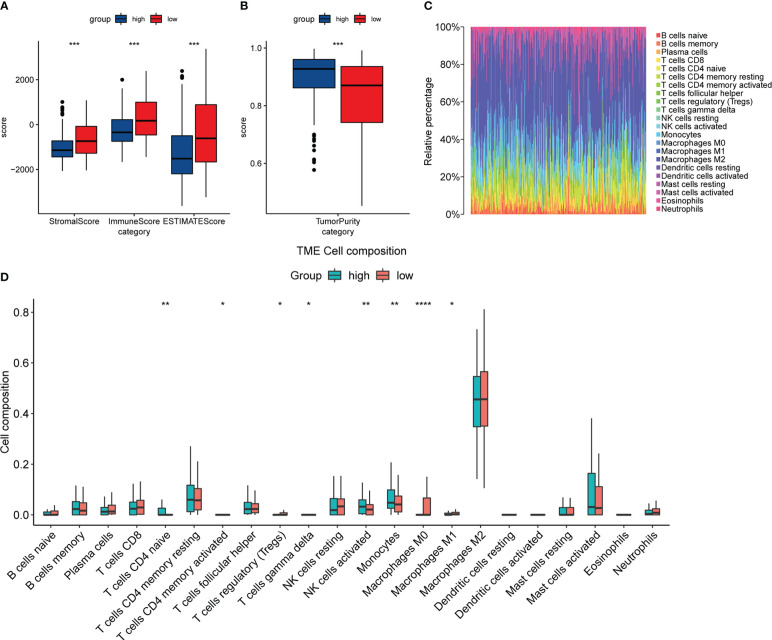
The relationship between the expression of DACH1 and the immune infiltration status. **(A)** The StromalScore, ImmuneScore, and ESTIMATEScore were significantly higher in the low-risk group than in the high-risk group (P < 0.05). **(B)** The TumorPurity was significantly lower in the low-risk group than in the high-risk group (P < 0.05). **(C)** The proportion of 22 immune cell types in the 188 patients in the training set. **(D)** The DACH1 low expression group had more regulatory T cells and fewer NK cells activated.

The “estimate” package and the Cibersortx website were used to analyze the expression matrix of patients treated with temozolomide in the train set. The results of the “estimate” package analysis showed that the DACH1 low expression group had higher StromalScore, ImmuneScore, and ESTIMATEScore, as well as lower tumor purity (**Figures 11A, B**), indicating that downregulating DACH1 can promote immune cell infiltration. The results of the Cibersortx website analysis showed the proportion of 22 immune cell types in 188 patients in the training set (**Figure 11C**) and the difference of 22 immune cell types between the DACH1 high expression group and low expression group (**Figure 11D**). There were more regulatory T cells and less activated NK cells in the DACH1 low expression group.

### The relationship between the expression of DACH1 and clinical features

**Figure 12:**
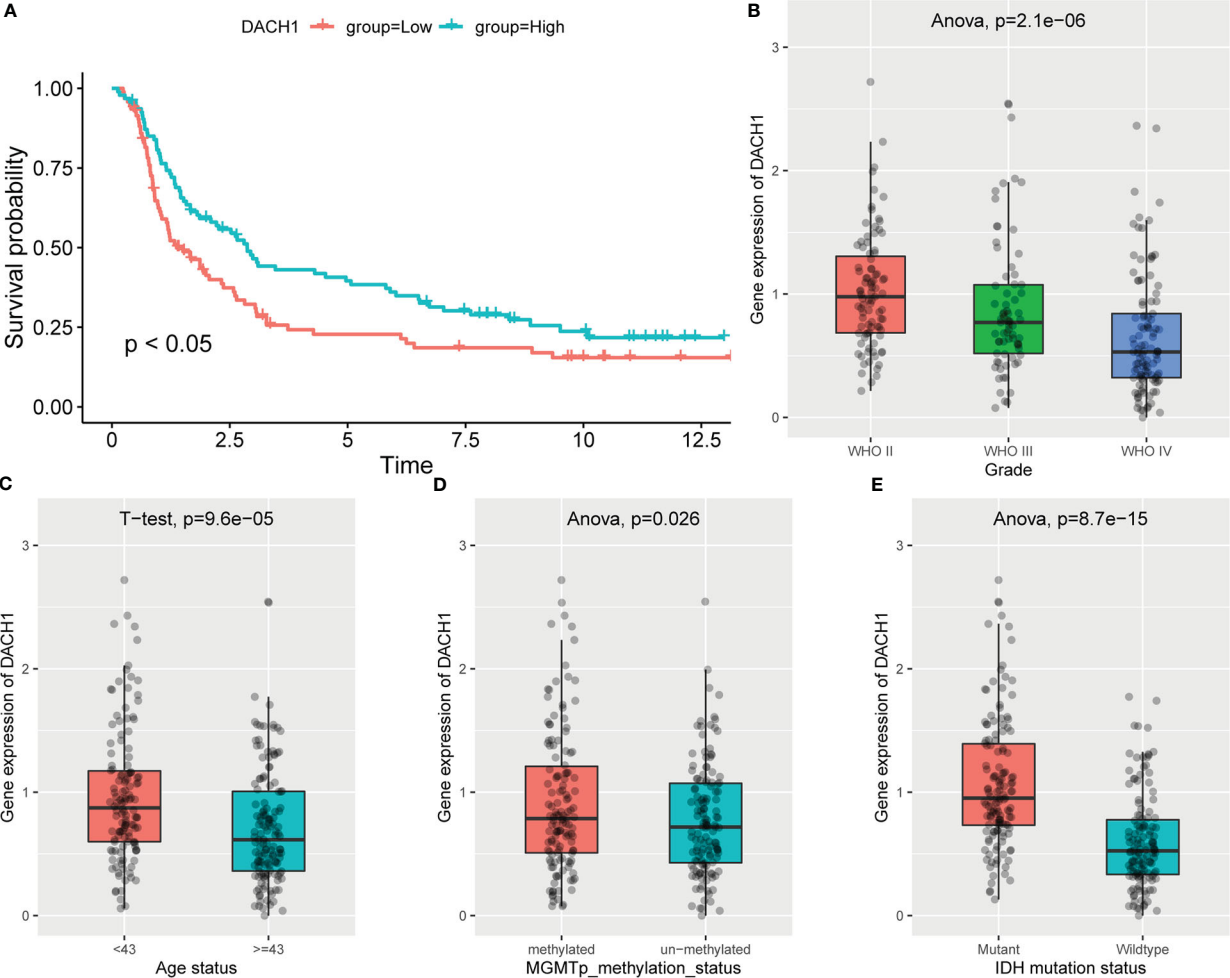
The relationship between the expression of DACH1 and clinical features. **(A)** Red indicates the DACH1 low expression group, and green indicates the DACH1 high expression group. Patients with low expression had a lower survival rate than those with high expression (P < 0.05). **(B)** The expression of DACH1 decreases with increasing glioma grade. **(C)** The expression of DACH1 in the >=43-year-old group was significantly lower than that in the <43-year-old group (P < 0.05). **(D)** The expression of DACH1 in the non-methylated group was significantly lower than that in the methylated group (P < 0.05). **(E)** The expression of DACH1 in the IDH wild-type group was significantly lower than that in the IDH mutant group (P < 0.05).

Patients treated with temozolomide were divided into high and low expression groups based on the median DACH1 expression, and the Kaplan-Meier method was used to analyze the survival difference. The results showed that the survival rate in the low expression group was significantly lower than that in the high expression group (P<0.05) (**Figure 12A**). The ANOVA (Analysis of Variance) and the t-test were used to analyze the relationship between DACH1 expression and tumor grade, patient age, MGMT methylation status, and IDH mutation status. The results show that as the glioma grade increases, DACH1 expression decreases (**Figure 12B**). The expression of DACH1 in the >=43-year-old group was significantly lower than that in the <43-year-old group (P < 0.05) (**Figure 12C**). The expression of DACH1 in the non-methylated group was significantly lower than that in the methylated group (P < 0.05) (**Figure 12D**). The expression of DACH1 in the IDH wild-type group was significantly lower than that in the IDH mutant group (P < 0.05) (**Figure 12E**).

## Discussion

Gliomas have great intra- and inter-tumoral heterogeneity (17), which makes both research and treatment very complex. Due to the limitations of histopathology, molecular biomarkers are becoming more and more critical in assisting with diagnosis, guiding treatment, and evaluating prognosis (18). Diffuse astrocytoma with TERT promoter mutations, EGFR amplification, and/or +7/-10 copy number variants in IDH wild type have biological behavior equivalent to glioblastoma (WHO grade 4), even though they exhibit histologically as WHO grade 2/3 (19); WHO grade 2/3 IDH-wild/H3-wild diffuse glioma with BRAF V600E mutation, FGFR1 variant, and/or MYB/MYBL1 variant are suggestive of biological indolence and have a better prognosis for patients (20). The 2021 WHO CNS5 has recognized molecular biomarkers as crucial signatures for glioma classification (21). Therefore, exploratory research on feature genes is increasingly important. In this study, gene expression information and clinical information of patients were obtained from the GEO, TCGA, and CGGA databases. Clinical data from temozolomide-treated patients was chosen to predict drug sensitivity, because the longer survival times of these patients suggest better sensitivity to the drug. A risk model for predicting glioma susceptibility to temozolomide was built based on the feature genes identified through differential analysis, PPI, machine learning, etc. The prediction results in both the training set and the validation set demonstrated that the risk model had good predictive ability. We then predict a number of potential compounds that could overcome temozolomide resistance by regulating the model signature genes, and we intend to further validate them in subsequent experiments. In the process of functional prediction of model signature genes, we found that DACH1 is closely associated with transcriptional misregulation in cancer and TGF-beta, both of which are crucial for the development of tumor treatment resistance. Therefore, we chose the DACH1 gene for our further experimental research. DACH1 was first discovered as a key gene involved in Drosophila retinal development (22). Further research found that DACH1 expression was down-regulated in a variety of cancers and was closely related to poor prognosis (23). DACH1 regulates the development and progression of liver cancer *via* the Wnt/-catenin pathway (24), promotes breast cancer growth and metastasis *via* cyclin D1 (25), and maintains glioma cell stemness *via* bFGF transcriptional activation (26). Loss of DACH1 causes tumor cells to proliferate and migrate, which has also been observed in prostate, kidney, and lung adenocarcinomas (27–29).

In this study, we chose the U251 cell line, which was derived from glioma patient tumor tissue and is one of the most commonly used cell lines in glioma research. We investigated the characteristics of the U251 cell line in more detail on the Cell Model Passports website (https://cellmodelpassports.sanger.ac.uk/) and found that DACH1 was wild type in the U251 cell line. Then, we observed the genetic changes after converting U251 into U251TR. The results of the western blot and qPCR experiments showed that the expression of DACH1 was significantly lower in U251TR. The sensitivity of U251 to temozolomide was lowered after DACH1 was silenced. The above results suggest that DACH1 is downregulated in U251TR and the silencing of DACH1 causes U251 to become resistant to temozolomide. In order to determine the function of DACH1 in temozolomide resistance, we ran GSEA and immune infiltration analysis. The GSEA results showed that transcriptional misregulation in cancer, ECM-receptor interaction, and the NF-kappa B signaling pathway were activated in the DACH1 low expression group. Transcriptional misregulation in cancer is an important player in regulating tumor development, metastasis, and chemotherapy resistance (30), and it was found to be closely related to DACH1 in the functional prediction (**Figure 5B**). Thus, DACH1 very likely regulates glioma temozolomide resistance through transcriptional misregulation in cancer. According to the GSEA results, the ECM-receptor interaction and NF-kappa B signaling pathways were also found to be active in the DACH1 low expression group, which is consistent with the research of Sattout Aman (31). We further confirmed this using the scratch assay. The proliferation of U251 cells was unaffected by temozolomide at the concentration of 50 μM, but their migration was restricted. However, after DACH1 down-regulation, temozolomide’s ability to prevent tumor migration was lost, and the cells even displayed a stronger capacity for invasion than in temozolomide-free conditions. The powerful invasive ability is probably a reason that tumors develop drug resistance. Therefore, DACH1 probably regulates these pathways to contribute to temozolomide resistance in glioma. The results of the immune infiltration analysis showed that patients in the DACH1 low expression group had higher immune scores and lower tumor purity, which suggested that DACH1 could be able to promote immune cell infiltration. The results of the online analysis on the Cibersortx website showed that the DACH1 low expression group had more regulatory T cells and fewer NK cells activated, suggesting that the downregulation of DACH1 could promote the infiltration of immunosuppressive types of cells, which is likely a mechanism that DACH1 regulates drug resistance.

Following a more in-depth investigation, we discovered that DACH1 expression was closely related to several clinical characteristics in patients. The survival rate of temozolomide-treated patients in the low DACH1 expression group was lower, indicating that DACH1 can affect prognosis. Furthermore, DACH1 expression decreased significantly with increasing tumor grade, and it was also lower in patients with advanced age, IDH wild-type, and MGMT non-methylation. Advanced age, higher grade, MGMT non-methylation, and IDH wild type are all poor prognostic factors in glioma patients (32). We found DACH1 mutations (mainly inframe deletions) and DNA methylation in some patients while investigating the Brain Tumor PDXs (Mayo Clinic, Clin Cancer Res 2020) dataset on the cbioportal website. These alterations were also taken into consideration as possible reasons for the downregulation of DACH1 in some patients. Further investigation revealed that the DACH1-altered group’s survival was significantly worse than that of the DACH1-unaltered group, which also matched our study.

## Conclusion

This study builds and verifies a risk model for temozolomide-treated patients to predict their prognosis and explores the function of one of the feature genes, DACH1, in gliomas.

This study provides a reliable model and a promising target for the research of temozolomide resistance.

## Data availability statement

The original contributions presented in the study are included in the article/**Supplementary material**. Further inquiries can be directed to the corresponding author.

## Author contributions

X-FW and QG conceived the study and constructed the study design. QG wrote the manuscript. LL and F-YD handled the partial data. JY, YG, SZ, and XW helped create the figures and tables. All authors accept responsibility for the work’s contents. All authors contributed to the article and approved the submitted version.
