# Supplementary figures and images for: Identification and verification of the temozolomide resistance feature gene DACH1 in gliomas
Source: Front Oncol. 2023 Mar 7;13:1120103. doi: 10.3389/fonc.2023.1120103 (PMC10028258; doi:10.3389/fonc.2023.1120103)

**U251**

**U251TR**

ns

ns

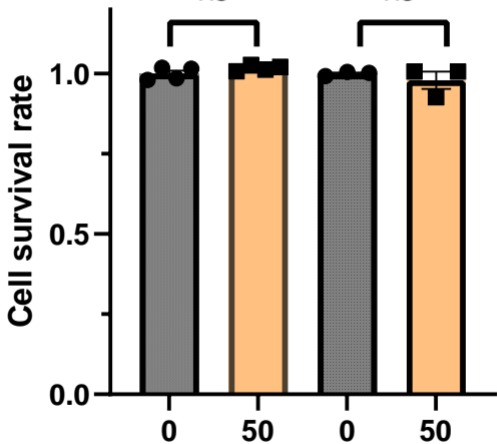

**Temozolomide concentration (μM)**

Supplement: Supplementary file 2 [file DataSheet_2.pdf]
